# A Quantitative Subarachnoid Hemorrhage Grading System, Including Supratentorial and Infratentorial Cisterns, With Multiplanar Computed Tomography Reformations

**DOI:** 10.7759/cureus.27025

**Published:** 2022-07-19

**Authors:** Einat Slonimsky, Tao Ouyang, Kent Upham, Sarah Pepley, Tonya King, Marco Fiorelli, Krishnamoorthy Thamburaj

**Affiliations:** 1 Diagnostic Radiology, Milton S. Hershey Medical Center, Hershey, USA; 2 Diagnostic Radiology, University of Rochester Medical Center, Rochester, USA; 3 Diagnostic Radiology, University of Pittsburgh Medical Center, Pittsburgh, USA; 4 Department of Biostatistics, Penn State Health College of Medicine, Hershey, USA; 5 Department of Human Neuroscience, Sapienza University of Rome, Rome, ITA

**Keywords:** subarachnoid hemorrhage, ct (computed tomography) imaging, spontaneous intracranial hemorrhage, cerebral vasospasm, computed tomography (ct )

## Abstract

Background

Subarachnoid hemorrhage (SAH) grading scales typically evaluate a limited number of cisterns on the axial plane. The goal of our study is to apply a simple quantitative yet comprehensive SAH grading scale to all major intracranial cisterns, including the infratentorial cisterns, with multiplanar computed tomography (CT) reformations.

Methodology

We performed a retrospective review of 94 consecutive cases of spontaneous SAH presenting within 72 hours of onset. SAH was categorized into five grades based on the short-axis thickness of SAH in 20 intracranial cisterns measured on the axial, coronal, and sagittal planes. Statistical analysis was performed for inter-rater agreement with kappa statistics, for inter-plane agreement by Spearman correlation statistics, and for inter-rater and inter-plane agreement by Pearson correlation statistics.

Results

The extended kappa coefficient for the three reviewers across all 20 cisterns varied from 0.38 (0.27, 0.50) to 0.59 (0.52, 0.65) on the axial plane. The kappa coefficient for two reviewers varied from 0.46 (0.33, 0.59) to 0.70 (0.60, 0.80) on the coronal plane and from 0.35 (0.20, 0.49) to 0.87 (0.77, 0.96) on the sagittal plane. The average grade of cisterns per case demonstrated mostly excellent correlation between the imaging planes with Spearman correlation statistics (≥0.70). Pairwise concordance correlation coefficient of the total SAH score revealed agreement ranging from 0.81 to 0.90 in all three planes. Pearson correlation statistics of the average total SAH scores revealed excellent correlation among the three planes (≥0.91).

Conclusion

A simple quantitative SAH grading scale can be successfully applied to the supratentorial and infratentorial cisterns in three standard CT imaging planes.

## Introduction

Several studies have found a correlation between the risk of vasospasm and the severity of subarachnoid hemorrhage (SAH) [1]. When more cisterns are included in the grading scale, vasospasm risk can be more accurately predicted [2]. Therefore, of the currently available SAH grading scales, it is not surprising that the Hijdra scale has demonstrated the best cerebral vasospasm prediction as it evaluates SAH in more cisterns [3,4]. Although the Hijdra scale grades SAH in 10 basal cisterns, similar to other scales, it excludes the grading of infratentorial cisterns [5-9]. SAH in infratentorial cisterns may originate from the rupture of posterior circulation aneurysms or extensions of the SAH from ruptured supratentorial aneurysms [10]. Because the risk of vasospasm is related to the extent of SAH, a grading scale that incorporates evaluation of SAH infratentorial cisterns would seem advantageous. The introduction of multiplanar computed tomography (CT) has led to improved identification of traumatic SAH compared to traditional axial plane CT images [11]. In this study, we used multiplanar CT to grade SAH in 20 major intracranial cisterns, double the number of cisterns evaluated by the Hijdra scale. We target all well-known aneurysm rupture sites and include infratentorial cisterns.

## Materials and methods

Participants

This retrospective study was conducted in compliance with the 1996 Health Insurance Portability and Accountability Act and approved by the Institutional Review Board (The Pennsylvania State University, STUDY00009671) that waived the patient consent requirement. Relevant consecutive cases were identified on our institutional database through our picture archiving and communication system (PACS) using the Primordial search engine (Primordial, Inc., California, USA). We used the search terms “subarachnoid” and “SAH” and set the date parameters as January 2007 to February 2018. The search yielded 215 consecutive cases of spontaneous SAH admitted to our emergency department. Inclusion criteria were patients diagnosed with SAH on CT within three days of onset and multiplanar coronal and sagittal CT reformations. In total, 94 consecutive cases were identified for inclusion in our study.

Image acquisition

Multidetector CT machines from different vendors were used in the cases studied. Images were acquired from the vertex to C2 vertebral level on the axial plane, with 3 to 5 mm slice thickness at zero spacing, 240 mm field of view, and a 512 × 512-pixel image matrix. Coronal and sagittal reformations were performed with 2 to 3 mm slice thickness at zero spacing.

Image interpretation

**Figure 1:**
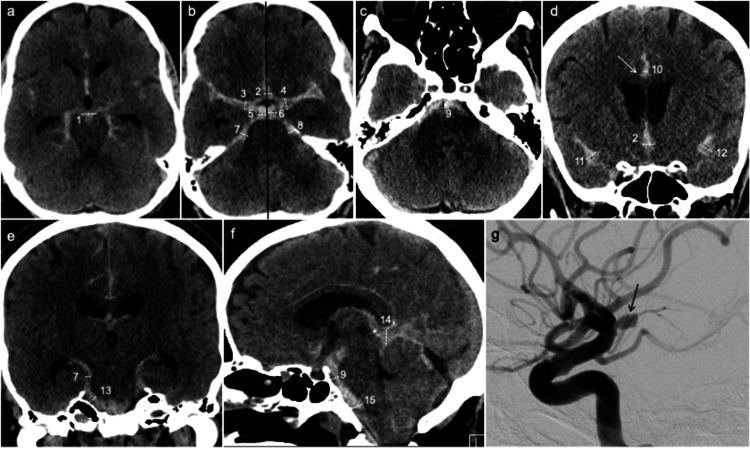
SAH from a ruptured aneurysm. CT scans of a 58-year-old male with acute SAH from a ruptured right carotid terminus aneurysm. The numbers in parentheses denote the cisterns. (a) Axial CT showing grade III 10-mm-thick SAH in the interpeduncular cistern (1). (b) Axial CT. The black central line demarcates the right and left sides of the suprasellar cisterns. The image shows a grade III 8 mm SAH in the ACom cistern (2), grade II 6 mm SAH in the base of the right Sylvian cistern (3), grade III 8 mm SAH in the base of the left Sylvian cistern (4), grade III 8 mm SAH in the right suprasellar cistern (5), grade II 6 mm SAH in the left suprasellar cistern (6), grade II 5 mm SAH in the right ambient cistern (7), and grade II 5 mm SAH in the left ambient cistern (8). (c) Axial CT showing a grade III 8 mm SAH in the prepontine cistern (9). (d) Coronal CT showing a grade III 8 mm SAH in the ACom cistern (2), grade I 3 mm SAH in the interhemispheric cistern (10), grade III 8 mm SAH in the right lateral Sylvian cistern (11), and grade III 8 mm SAH in the left lateral Sylvian cistern (12). Thin extension of a SAH into sulci branching from the main cistern. This is not included in the measurement of SAH thickness (arrow). (e) Coronal CT showing a grade II 4 mm SAH in the right ambient cistern (7) and a grade II 5 mm SAH in the right cerebellopontine angle cistern (13). (f) Sagittal CT showing a grade III 8 mm SAH in the prepontine cistern (9), a grade III 9 mm SAH in the quadrigeminal cistern (14), and a grade III 7 mm SAH in the premedullary cistern (15). (g) DSA of the same patient of a selective lateral view of the right internal carotid artery shows an irregular, posteriorly projecting 4 mm saccular aneurysm at the right carotid terminus (arrow). SAH: subarachnoid hemorrhage; CT: computed tomography; ACom: anterior communicating artery; DSA: digital subtraction angiography

CT images were independently analyzed on the PACS by three fellowship-trained neuroradiologists (two neuroradiologists with more than 10 years of experience and a graduate neuroradiology fellow) who were blinded to the clinical data, radiology reports, and all angiographic images, including CT angiography and digital subtraction angiography. The images on the axial plane were analyzed by all three reviewers (R1, R2, and R3) and in the sagittal and coronal planes by two reviewers (R1 and R2). The maximum thickness of SAH in the shortest axis of a cistern in each imaging plane was measured and categorized into one of five grades: 0, no SAH; I, ≤3 mm; II, >3 to ≤6 mm; III, >6 to ≤11 mm; and IV, >11 mm (Figure 1). The grading scale was used to analyze 20 major intracranial cisterns per patient, per imaging plane, including 12 supratentorial and eight infratentorial cisterns. This included all well-known locations of ruptured intracranial aneurysms (Table 1). To avoid overestimating SAH thicknesses, measurements excluded thin linear extensions of SAH from the main cistern into adjacent sulci. Near the suprasellar cistern, a midline demarcation was used to measure the width of hemorrhages in the right and left lateral suprasellar cisterns. When it was not possible to measure the short axis of a SAH due to the orientation of a cistern in an imaging plane or another cause, the measurement was documented as “difficult to assess” (DA). To familiarize the reviewers with the grading system, they initially analyzed the CT of five cases separate from the study cohort. Images in one plane from four or five cohort cases per day were read in a randomized order by each reviewer. In each case, images from each plane for each case were read on different days to those from the other two planes for that case to avoid association bias. In each case, the grading per plane required approximately one to two minutes to complete.

**Table 1:** The relationships of 20 major intracranial cisterns to aneurysm rupture sites, including the infratentorial (numbered 1 to 8) and supratentorial cisterns (numbered 9 to 20). A1: main stem of the anterior cerebral artery; AChA: anterior choroidal artery; ACom: anterior communicating artery; AICA: anterior inferior cerebellar artery; ASS: anterior suprasellar cistern; BA: basilar artery; CM: cisterna magna; CT: carotid terminus; IHF: interhemispheric fissure; IP: interpeduncular cistern; LAC: left ambient cistern; LBS: left basal Sylvian cistern; LCM: left cerebellomedullary cistern; LCPA: left cerebellopontine angle cistern; LLS: left lateral Sylvian cistern; LSS: left suprasellar cistern; M1: main stem of the middle cerebral artery; MCA: middle cerebral artery; OICA: ophthalmic segment internal carotid artery; PCA: posterior cerebral artery; PCom: posterior communicating artery; PICA: posterior inferior cerebellar artery; PM: premedullary cistern; PMSAH: perimesencephalic subarachnoid hemorrhage; PP: prepontine cistern; QC: quadrigeminal cistern; RAC: right ambient cistern; RBS: right basal Sylvian cistern; RCM: right cerebellomedullary cistern; RCPA: right cerebellopontine angle cistern; RLS: right lateral Sylvian cistern; RSS: right suprasellar cistern; SCA: superior cerebellar artery; VA: vertebral artery

	Cisterns	Aneurysm relation
1	C1-C2 cistern	PICA
2	CM	PICA
3	RCM	Right VA-PICA junction
4	LCM	Left VA-PICA junction
5	PM	VA-BA junction
6	RCPA	Right AICA or SCA
7	LCPA	Left AICA or SCA
8	PP	BA; PMSAH
9	IP	BA tip; PMSAH
10	RAC	Right PCA, SCA, or PMSAH
11	LAC	Left PCA, SCA, or PMSAH
12	RSS	Right OICA, PCom, or AChA
13	LSS	Left OICA, PCom, or AChA
14	ASS	ACom
15	RBS	Right CT, M1, or A1
16	LBS	Left CT, M1, or A1
17	RLS	Right MCA bifurcation
18	LLS	Left MCA bifurcation
19	IHF	DACA (anterior), PCA (posterior)
20	QC	PCA or PMSAH variant

Statistical analysis

Inter-rater agreement (IRA) on the grades was assessed by estimating the kappa and extended kappa coefficients between the reviewers. Kappa <0 was rated as below-chance agreement, 0.01-0.20 as slight agreement, 0.21-0.40 as fair agreement, 0.41-0.60 as moderate agreement, 0.61-0.80 as substantial agreement, and 0.81-0.99 as almost perfect agreement [12]. Agreement on the coronal and sagittal planes between two reviewers was evaluated with the kappa coefficient for each of the 20 individual cisterns. Agreement on the axial plane between the three reviewers was evaluated with the extended kappa coefficient for each of the 20 individual cisterns. The grades were then averaged per cistern per case per plane and the correlation was estimated among the planes for each cistern separately using Spearman’s correlation coefficient. 95% confidence intervals (CI) based on Fisher’s z transformation were reported. Each total SAH score was obtained by tallying the SAH grades of the reviewers for each plane. Agreement among reviewers for the total score was assessed for each plane using pairwise concordance correlation coefficients because the distribution of the total score was approximately normal for each plane and reviewer. Finally, the total scores for each reviewer were averaged to obtain a total score for each plane for each case. The correlation between the total scores of the three imaging planes was assessed using Pearson’s correlation coefficient with 95% CI based on Fisher’s z transformation. The sizes of correlation coefficients were interpreted as very high positive (0.90-1.00), high positive (0.70-0.90), moderate positive (0.50-0.70), low positive (0.30-0.50), or negligible (0.00-0.30) [13]. Statistical analyses were performed using SAS statistical software version 9.4 (SAS Institute Inc., Cary, NC, USA).

## Results

A total of 94 cases (male-to-female ratio, 27:67) with an average age of 54.6 (range 13-81 years, SD ± 12.1) were evaluated. In total, 1,880 cisterns were assessed per imaging plane. Overall, 13,160 cisterns were assessed by the reviewers, 827 (6%) of which were documented as DA. The prepontine cistern was documented as DA on the coronal plane 82 and 71 times by R1 and R2, respectively. On the coronal plane, the premedullary cistern was documented as DA 74 and 46 times by R1 and R2, respectively, and the C1-C2 cistern was documented as DA 60 times by R1. This resulted in insufficient data for these three cisterns on the coronal plane. On the sagittal plane, the right cerebellomedullary cistern was scored as DA in 50 cases and the left in 46 cases by R1. On the same plane, the anterior suprasellar cistern was scored as DA in 62 cases and the interhemispheric fissure in 79 cases by R2. The axial plane allowed grading of all cisterns by the three reviewers without difficulty. Statistical analyses of the cerebellomedullary cisterns, anterior suprasellar cistern, and interhemispheric fissure on the sagittal plane were obtained with sparse data.

The results of the IRA on the axial plane between the three reviewers (R1, R2, and R3) and on the coronal and sagittal planes between the two reviewers (R1 and R2) are shown in Table 2. The extended kappa coefficient for the three reviewers across all 20 cisterns varied from 0.38 (0.27, 0.50) to 0.59 (0.52, 0.65) on the axial plane. There was moderate reviewer agreement on the axial plane for 19 of the cisterns, with IRA ranging from 0.43 (0.32, 0.43) to 0.57 (0.51, 0.62) for all but the interpeduncular cistern. SAH grading of the interpeduncular cistern on the axial plane revealed only fair agreement, with an IRA of 0.38 (0.27, 0.50). On the axial plane, the IRA for the supratentorial and infratentorial cisterns ranged from 0.43 (0.32, 0.43) to 0.57 (0.51, 0.62) and from 0.40 (0.43, 0.54) to 0.59 (0.52, 0.65), respectively. The kappa coefficient on the coronal plane for the two reviewers varied from 0.45 (0.25, 0.65) to 0.70 (0.60, 0.80). There was substantial agreement on 9/20 cisterns on the coronal plane. The kappa coefficient on the sagittal plane for the two reviewers varied from 0.35 (0.20, 0.49) to 0.87 (0.77, 0.96). There was fair agreement on the interpeduncular cistern on the sagittal plane (0.35 (0.20, 0.49)). There was substantial agreement regarding the eight other cisterns on the sagittal plane, with IRA ranging from 0.61 (0.50, 0.72) to 0.87 (0.77, 0.96), although 4/8 of these cisterns provided sparse data. Among the 60 cisterns evaluated on the three planes, there were only two instances of fair agreement between the reviewers. This was for the interpeduncular cistern on the axial and sagittal planes. With the exclusion of these two cisterns and the three cisterns on the coronal plane for which there was insufficient data, there was moderate to substantial agreement regarding the remaining 55 cisterns, with IRA ranging from 0.41 (0.31, 0.51) to 0.70 (0.60, 0.80) (Table 2). Spearman’s correlation coefficients for an average grade SAH in each cistern per plane produced, overall, substantial to almost perfect positive correlations between the planes, ranging from 0.62 (0.37-0.79) to 0.89 (0.83-0.92). It revealed a moderate positive correlation between the cisterns and planes in seven instances out of 60 comparisons, overall, with at least one comparison between the planes yielding a substantial to almost perfect positive correlation (Table 3). Pairwise concordance correlation coefficients of the total SAH score were obtained by tallying all the grades for the reviewers on all three planes. This revealed high positive correlations, ranging from 0.81 to 0.90 (Table 4). Pearson’s correlation coefficients of the averages of the total scores were obtained by tallying the SAH grades. This revealed very high positive correlations, ranging from 0.91 to 0.93, between the three imaging planes (Table 5).

**Table 2:** Inter-rater agreement on the grading of individual cisterns in each of the three computed tomography imaging planes. *Based on sparse data. CM: cisterna magna; RCM: right cerebellomedullary cistern; LCM: left cerebellomedullary cistern; PM: premedullary cistern; RCPA: right cerebellopontine angle cistern; LCPA: left cerebellopontine angle cistern; PP: prepontine cistern; IP: interpeduncular cistern; RAC: right ambient cistern; LAC: left ambient cistern; RSS: right suprasellar cistern; LSS: left suprasellar cistern; ASS: anterior suprasellar cistern; RBS: right basal Sylvian cistern; LBS: left basal Sylvian cistern; RLS: right lateral Sylvian cistern; LLS: left lateral Sylvian cistern; IHF: interhemispheric fissure; QC: quadrigeminal cistern

		Extended kappa (three reviewers)	Kappa coefficient (two reviewers)	
	Cisterns	Axial	Coronal	Sagittal
1	C1-C2	0.53 (0.47, 0.60)	Insufficient data	0.64 (0.52, 0.76)
2	CM	0.52 (0.41, 0.63)	0.62 (0.48, 0.77)	0.61 (0.50, 0.72)
3	LCM	0.59 (0.52, 0.65)	0.54 (0.39, 0.70)	0.87 (0.77, 0.96)*
4	RCM	0.58 (0.53, 0.63)	0.67 (0.55, 0.80)	0.73 (0.61, 0.86)*
5	PM	0.48 (0.43, 0.54)	Insufficient data	0.56 (0.45, 0.66)
6	LCPA	0.50 (0.44, 0.55)	0.60 (0.48, 0.72)	0.51 (0.39, 0.63)
7	RCPA	0.54 (0.49, 0.59)	0.56 (0.44, 0.69)	0.51 (0.40, 0.61)
8	PP	0.53 (0.48, 0.58)	Insufficient data	0.59 (0.45, 0.72)
9	PSS	0.38 (0.27, 0.50)	0.45 (0.25, 0.65)	0.35 (0.20, 0.49)
10	RAC	0.43 (0.32, 0.54)	0.55 (0.44, 0.66)	0.52 (0.41, 0.64)
11	LAC	0.50 (0.44, 0.57)	0.51 (0.39, 0.64)	0.46 (0.32, 0.59)
12	RSS	0.50 (0.45, 0.56)	0.63 (0.53, 0.73)	0.49 (0.37, 0.61)
13	LSS	0.55 (0.50, 0.60)	0.67 (0.59, 0.75)	0.49 (0.37, 0.60)
14	ASS	0.55 (0.50, 0.61)	0.47 (0.35, 0.58)	0.77 (0.64, 0.91)*
15	RBS	0.57 (0.51, 0.62)	0.70 (0.60, 0.80)	0.62 (0.51, 0.73)
16	RLS	0.55 (0.50, 0.60)	0.62 (0.51, 0.73)	0.49 (0.39, 0.58)
17	LBS	0.50 (0.43, 0.56)	0.60 (0.50, 0.70)	0.52 (0.42, 0.63)
18	LLS	0.53 (0.48, 0.59)	0.60 (0.49, 0.71)	0.41 (0.31, 0.51)
19	IHF	0.51 (0.44, 0.57)	0.53 (0.42, 0.65)	0.72 (0.50, 0.93)*
20	QC	0.53 (0.45, 0.60)	0.46 (0.33, 0.59)	0.63 (0.52, 0.74)

**Table 3:** Correlations between the three computed tomography imaging planes for average grading of subarachnoid hemorrhages in cisterns per case, obtained using Spearman’s correlation coefficient. R1: Reviewer 1; R2: Reviewer 2; R3: Reviewer 3; CM: cisterna magna; RCM: right cerebellomedullary cistern; LCM: left cerebellomedullary cistern; PM: premedullary cistern; RCPA: right cerebellopontine angle cistern; LCPA: left cerebellopontine angle cistern; PP: prepontine cistern; IP: interpeduncular cistern; RAC: right ambient cistern; LAC: left ambient cistern; RSS: right suprasellar cistern; LSS: left suprasellar cistern; ASS: anterior suprasellar cistern; RBS: right basal Sylvian cistern; LBS: left basal Sylvian cistern; RLS: right lateral Sylvian cistern; LLS: left lateral Sylvian cistern; IHF: interhemispheric fissure; QC: quadrigeminal cistern

Cistern	Number of cases (R1, R2, R3)	Axial vs. coronal	Axial vs. sagittal	Sagittal vs. coronal
C1-C2	91, 92, 89	0.57 (0.41–0.69)	0.78 (0.68–0.85)	0.57 (0.41–0.70)
CM	94, 93, 93	0.77 (0.67–0.84)	0.84 (0.77–0.89)	0.77 (0.67–0.84)
LCM	94, 90, 90	0.71 (0.59–0.80)	0.77 (0.67–0.84)	0.73 (0.62–0.82)
RCM	94, 90, 90	0.74 (0.63–0.82)	0.76 (0.66–0.84)	0.74 (0.63–0.82)
PM	55, 94, 55	0.48 (0.25–0.66)	0.83 (0.75–0.88)	0.47 (0.24–0.65)
LCPA	94, 93, 93	0.83 (0.76–0.88)	0.74 (0.63–0.82)	0.78 (0.69–0.85)
RCPA	94, 93, 93	0.84 (0.77–0.89)	0.81 (0.72–0.87)	0.78 (0.69–0.85)
PP	27, 94, 27	0.30 (-0.09–0.61)	0.74 (0.63–0.82)	0.27 (-0.12–0.59)
PSS	36, 94, 36	0.62 (0.37–0.79)	0.43 (0.25–0.58)	0.46 (0.15–0.69)
RAC	94, 94, 94	0.86 (0.80–0.90)	0.83 (0.76–0.89)	0.85 (0.78–0.89)
LAC	94, 94, 94	0.77 (0.67–0.84)	0.79 (0.70–0.86)	0.82 (0.74–0.88)
RSS	94, 94, 94	0.73 (0.61–0.81)	0.78 (0.69–0.85)	0.83 (0.76–0.88)
LSS	94, 94, 94	0.80 (0.71–0.86)	0.83 (0.76–0.89)	0.79 (0.69–0.85)
ASS	94, 92, 92	0.79 (0.70–0.85)	0.67 (0.53–0.77)	0.62 (0.48–0.73)
RBS	94, 94, 94	0.83 (0.75–0.88)	0.77 (0.68–0.84)	0.84 (0.76–0.89)
RLS	94, 94, 94	0.89 (0.83–0.92)	0.84 (0.77–0.89)	0.87 (0.80–0.91)
LBS	94, 94, 94	0.74 (0.63–0.82)	0.80 (0.71–0.86)	0.83 (0.76–0.89)
LLS	94, 94, 94	0.85 (0.78–0.90)	0.77 (0.67–0.84)	0.86 (0.80–0.91)
IHF	94, 94, 94	0.82 (0.74–0.87)	0.74 (0.63–0.82)	0.76 (0.66–0.84)
QC	94, 94, 94	0.80 (0.72–0.87)	0.83 (0.75–0.88)	0.79 (0.70–0.86)

**Table 4:** Concordance correlation coefficients for inter-rater agreement on total subarachnoid hemorrhage scores, obtained by tallying all subarachnoid hemorrhage grades for each computed tomography imaging plane. R1: Reviewer 1; R2: Reviewer 2; R3: Reviewer 3; N/A: not applicable

Imaging plane	R1 vs. R2	R1 vs. R3	R2 vs. R3
Axial	0.90 (0.85, 0.93)	0.89 (0.84, 0.92)	0.81 (0.75, 0.87)
Coronal	0.89 (0.84, 0.92)	N/A	N/A
Sagittal	0.87 (0.81, 0.91)	N/A	N/A

**Table 5:** Pearson’s correlation coefficients for average summary scores, obtained by tallying the subarachnoid hemorrhage grades from the three computed tomography imaging planes.

Imaging plane comparison	Correlation estimates with 95% confidence interval
Axial vs. coronal	0.93 (0.90, 0.95)
Axial vs. sagittal	0.93 (0.89, 0.95)
Coronal vs. sagittal	0.91 (0.87, 0.94)

## Discussion

The risk of cerebral vasospasm is related to the severity of SAH [1,13]. Traditionally, SAH scales, such as the Hijdra scale, do not grade SAH in the infratentorial cisterns [5-9]. The new grading scale presented in this study uses a simplified quantitative measure and has shown that SAH in infratentorial cisterns can be graded successfully. The grading scale focuses on 20 major intracranial cisterns associated with well-known sites of aneurysm rupture. The scale showed substantial to almost perfect correlations for summary SAH score and average grading of cisterns between the three grading planes. For individual cisterns, there was primarily moderate IRA for cisterns on the axial plane and many cisterns on the coronal and sagittal planes. The incorporation of multiplanar CT images in SAH grading can provide improved information, as shown in Table 2.

The Hijdra scale has produced considerable interobserver variation when used to grade individual cisterns [14]. However, total SAH scores on the Hijdra scale produce better IRA than the grading of individual cisterns [5]. In the assessment of cerebral vasospasm risk, the total SAH scores produced by tallying Hijdra scale grades demonstrate better prediction of the risk of vasospasm than grades from individual cisterns [3,4,9,14]. Further, it has served as an independent predictor of delayed cerebral ischemia based on vasospasms and clinical outcomes [3,4]. Our study demonstrated comparable results with better IRA than that obtained by grading of individual cisterns; however, we incorporated infratentorial cisterns for a more comprehensive evaluation.

CONSCIOUS 1 to 3 trials evaluating the efficacy of the endothelin receptor antagonist, clazosentan, in the treatment of cerebral vasospasm, rely on simple quantitative measurements of variables such as the thickness and length of SAH in a cistern rather than using qualitative grading scales [15-17]. Measurement of the short-axis thicknesses of SAH using our scale permits a similar approach and allows evaluation of the extent and severity of SAH in all major intracranial cisterns, including the often-neglected infratentorial cisterns.

There were a few challenges to grading SAH with our scale. Difficulties occurred in measuring the short thickness of SAH in a very small number of cisterns on certain planes. This limitation was seen when grading the prepontine, premedullary, and C1-C2 cisterns on the coronal plane because their long axes precluded short-axis measurement of the SAH thickness. Similarly, on the sagittal plane, sparse grading data was available for the anterior suprasellar, interhemispheric, and bilateral cerebellomedullary cisterns. However, in all these challenging instances, grading was accomplished on at least one plane.

Some studies have categorized SAH with a thickness of >4 mm as “thick SAH clots.” Observation of “thick SAH clots” in three or more cisterns was used to distinguish diffuse SAH and successfully predict the risk of vasospasm-induced morbidity and outcomes [2,18]. Unlike the majority of qualitative SAH scales, the design of our scale is well suited to the additional measurement of the short-axis thicknesses of SAH and clots in as many cisterns as desired, with multiplanar CT reformations [5-7,9].

The retrospective design was a major limitation of our study. Artificial intelligence (AI) is increasingly used in imaging and, in the future, may minimize or eliminate the need for manual grading of SAH. However, current automated techniques incorporate several manual steps in the evaluation of SAH, and none is yet well accepted in clinical practice [19,20]. To date, no widely generalizable AI program has been developed for medical image analysis [21]. It is not known how long it will be before a reliable AI-dependent SAH scale is established. Further, the influence of the range of blood attenuations in evolving SAH and hematocrit levels, partial volume effects, and streak artifacts on the success of SAH grading with AI software is yet to be seen [20]. Our scale permits the evaluation of all major cisterns linked to common aneurysm rupture sites, including the infratentorial cisterns. Therefore, the comprehensiveness of our scale may serve to test the success of AI-based SAH grading. The exclusion of intraventricular hemorrhages (IVH) from grading was another important limitation of our scale. However, studies have demonstrated more consistent associations between the extent of cisternal SAH and vasospasm [22,23]. Research into the influence of IVH on the development of vasospasm has produced inconsistent results [9,19,20,24].

## Conclusions

We have presented a simple quantitative SAH grading scale using multiplanar CT scan images that can be successfully applied to the supratentorial and infratentorial cisterns on the three standard CT imaging planes. The proposed grading scale addresses a major shortcoming of previous scales with its inclusion of infratentorial cisterns. Our quantitative method of grading SAHs uses axial, coronal, and sagittal multiplanar CT reformations and is practical to apply in clinical settings. It offers a strong IRA for the overall grading of the extent of SAHs.
